# Can cystic lesions of the jaws be considered as the cause of mandibular asymmetry?

**DOI:** 10.4317/medoral.25134

**Published:** 2022-02-20

**Authors:** Mevlüde Polat, Onur Odabası

**Affiliations:** 1 Phd. Orcid: 0000-0001-9466-8447. Department of Orthodontics, Faculty of Dentistry, Harran University, 63300, Sanlıurfa, Turkey; 2 Phd. Orcid: 0000-0001-7771-048X. Department of Oral and Maxillofacial Surgery, Faculty of Dentistry, Ankara Yildirim Beyazit University, 06010, Ankara, Turkey

## Abstract

**Background:**

The aim of this study is to investigate the presence of condylar and ramal asymmetry in patients with a cyst larger than 10 mm in the maxilla or mandible.

**Material and Methods:**

Condylar and ramal asymmetry index measurements of 47 patients (mean age: 28.85 ± 15.348) in the study group and 40 patients in the control group (mean age: 33.73 ± 13.095) were performed using panoramic radiographs. The study group consists of patients with cysts larger than 10 mm in diameter in the maxilla or mandible. The control group consisted of patients with no radiolucent lesions and no history of trauma. The possible statistical difference between the groups was evaluated by the Mann-Whitney U test.

**Results:**

No statistically significant difference was observed in asymmetry indices according to gender and the jaw (maxilla or mandible) in which the cyst was located. However, it was determined that CAI and RAI values were statistically significantly different between the study and control groups (*p* = 0.047 and *p* = 0.016, respectively).

**Conclusions:**

The presence of intraosseous cysts larger than 10 mm in the jaws was found to be associated with condylar and ramal asymmetry.

** Key words:**Condylar asymmetry, ramal asymmetry, odontogenic cysts

## Introduction

Cysts are defined as a pathological cavity surrounded by epithelium-lined connective tissue capsule with a liquid or semi-solid material inside. Intra-bony cysts, with rare exceptions, are seen only in the jaw bones, as they contain a large number of dental epithelial remnants remaining after tooth development. Cysts originating from such dental epithelial remnants are called "odontogenic cysts" and classified as inflammatory and developmental cysts according to their etiology (1,2).

Odontogenic cysts grow slowly and can reach large sizes. Cysts may cause resorption and displacement of the tooth roots in the region where they develop, delayed tooth eruption, expansion and destruction of the bone (3). Thus, cysts cause changes on the biomechanical properties of the jaws, especially when they reach large sizes (4). In addition, caries, fractures and non-vital teeth associated with radicular cysts (5) cause changes on the chewing pattern (6,7).

Non-symmetrical structural disorders and functional imbalances in the jaws cause different growth and shaping of the right and left sides of the mandible and especially the condyle (8-10) Habets *et al*. identified a method in which the vertical heights of the right and left condyles were compared in panoramic radiographs to detect asymmetry between condyles and used this method as a diagnostic criterion for TMD (11). In the following years, the relationship of condylar asymmetry with TMJ disorders (12), occlusion types (8,13), posterior crossbite (9,14), parafunction (15) and occlusal asymmetry (10) has been investigated in many studies.

However, to the best of our knowledge, no attempt have been made to determine condylar and ramal asymmetry scores of the patients having both mandibular and maxillar odontogenic, intrabony cysts. Hereby, this study was intended to investigate whether the odontogenic cyst lessions have an effect on condylar and ramal asymmetry scores comparing healthy ones.

## Material and Methods

47 patients (22 males, 25 females, between the ages of 4-67) with an intra-osseous cyst larger than 10 mm in the panoramic radiography of the maxilla or mandible were included in the study group. The control group consisted of 40 patients (7 males, 33 females, 15-64 years old) without any cysts or degenerative conditions on their panoramic radiography. Subjects with insufficient demographic information or unsuiTable panoramic shots were not evaluated in this study. Ethics committee approval was obtained from Harran University Clinical Research Ethics Committee with the decision numbered HRU / 21.10.33. The PaX-I (Vatech, South Korea) machine with mA= 8, kVp = 60, and time = 10 s was used to obtain the panoramic images and this images were evaluated via EasyDent software. (Ver. 4.1.5.9)

**Figure 1:**
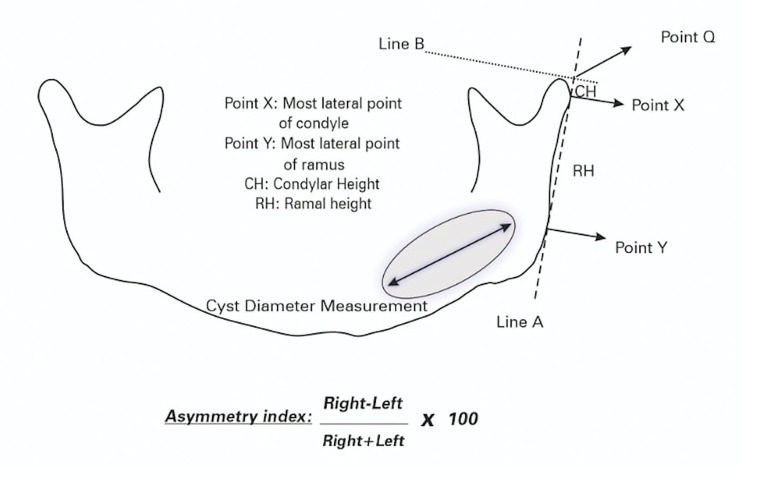
Measuring method described in Habets *et al*. and cyst diameter.

For right and left sides, the most external points of the condyle and ramus were mark as X and Y respectively. A refference line which called A were drawn between points X and Y. Then a second line B was drawn from the most superior of the condyles perpendicularly. The intersection of these lines (lines A and B) was recognised as point Q. (Fig. 1) The distance between lines Q and X measured and recorded as condylar height (CH) and distance between lines X and Y measured and recorded as ramal height (RH). As for the diameter measurement of cysts, the longest cyst diameter that can be measured, regardless of whether it is vertical or horizontal, was recorded and evaluated. The drawings and measurements were made by the same autor as blind assesment. Asymmetry in condylar and ramal heights were calculated with the formula introduced by Habets *et al*. (Fig. 1).

- Statistically analyse

The SPSS ver. 20.0 (IBM, NY, USA) was used for statistical analyses in the present study. Descriptive statistics were calculated and the significance level was set at 0.05. The variables condylar asymmetry index (CAI) and ramal asymmetry index (RAI) were evaluated by the Kolmogorov-Smirnov test and were not distributed normally. Therefore, the Mann-Whitney U test was used to test whether there was a significant difference between the two groups (study and control groups), jaws containing the cyst (maxilla and mandible), and genders.

## Results

The study sample consisted of 87 panoramic radiographs of the patients; 47 having 10.0 mm and above cycts size and 40 control. The mean cyst diameter was calculated as 33.46 mm ± 16.14. The mean age was 28,85 ± 15,34 for the study group, and 33,73 ± 13,095 for the control group. No significant difference among the groups was detected (Table 1).

Mandibular asymmetry indexes are shown in Table 2 separately for men and women in both groups. No significant difference was found between any of the variables according to gender.

When the asymmetry indexes were compared according to the study group and the control group, a significant difference was observed between the groups in CAI and RAI (Table 3).

There was no statistically significant difference between these two jaw groups when the CAI and RAI data of the cyst group were compared in terms of the occurrence of cysts in the maxilla or mandible (Table 4).

**Table 1:**
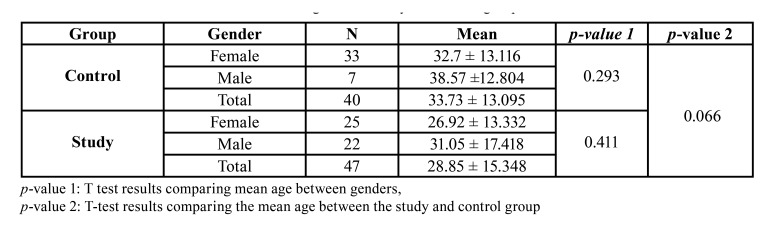
Means and standard deviations of the ages in the study and control groups.

**Table 2:**
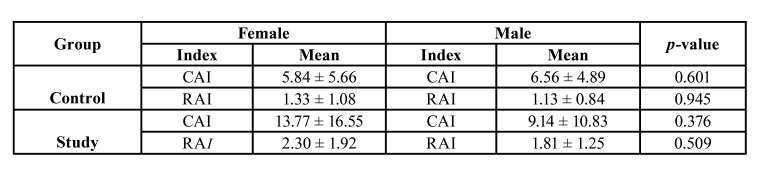
Descriptive statistics and comparisons of asymmetry indexes in the study and control groups according to gender.

**Table 3:**
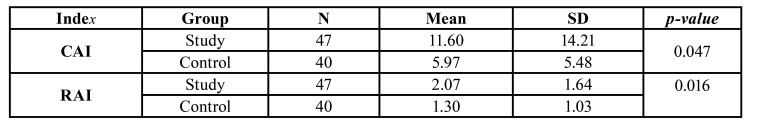
Comparison of the study group and the control group asymmetry index values.

**Table 4:**
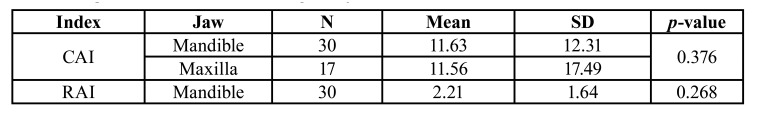
Comparison of CAI and RAI according to the jaws.

## Discussion

Intra-bony odontogenic cysts are very common pathologies of the oral and maxillofacial region. As a matter of fact, in a retrospective study of 739 patients (aged 4-84) who were treated for any lesion in the oral and maxillofacial region, it was reported that 63.19% of the lesions were odontogenic cysts (16). In this sense, it is important to examine the direct and indirect effects of cysts on the jaws.

There are many studies in the literature evaluating the relation of condylar asymmetry with TMJ disorders (12) , occlusal asymmetry (10), unilateral and bilateral posterior crossbite (9,14) and occlusion types (8). However, to the best of our knowledge, the relationship between condylar asymmetry and intra-bony cysts in the jaws has not been evaluated before.

Therefore, in our study, we aimed to determine whether intra-bony cysts in the maxilla or mandible cause condylar asymmetry. We designed this study on the idea that the effectiveness of chewing in patients with jaw cysts may be less used in chewing on the cyst-forming, expansed side. We used the method suggested by Habets *et al*. (11) on panoramic radiographs to investigate this hypothesis. The main reason for using panoramic radiographs for vertical mandibular asymmetry assessments is that these radiographs are easily accessible, their use is widespread in dentistry routine, and most importantly, bilateral imagination of the condylar and ramal portions of the mandible which are used to define the asymmetry index can be evaluated easyly (11,17).

Although there are vertical and horizontal magnifications on panoramic radiography, these magnifications are uniform and therefore do not affect the diagnosis (18). In addition, the source of vertical magnifications depends only on projection factors. The distance between the focal point of the X-ray tube and the film is always the same (19). Therefore, if the patient positioning is standardized, the statistical effect of magnification in the vertical direction between patients will be eliminated. As a matter of fact, in our study, maximum attention was paid to the positioning of the patients while taking panoramic radiography.

When we compared CAI and RAI according to gender in both control and study groups, we found that there was no statistically significant difference. This result was consistent with the findings of Habets *et al*. (11), Kiki *et al*. (20), Sezgin *et al*. (21), Tancan *et al* (14).

30 of the cysts in the study group are located in the mandible and 17 are in the maxilla. When the asymmetry index of the cysts was evaluated according to the jaw, no statistically significant difference was found in CAI and RAI. According to this result, the effect of the cysts on the condyle is independent of which jaw they are located in.

According to Habets, a 10 mm change in the head position when taking a panoramic x-ray can cause an asymmetry index of 3%. For this reason, an index value greater than 3% has been accepted as vertical asymmetry. In our study, CAI was measured as 5.97 ± 5.48 in the control group. Although this value indicates the presence of asymmetry, they measured CAI as Uysal *et al*. (14) 7.57 ± 8.39, Saglam *et al*. (22) 7.96 ± 6.73, Halıcıoğlu *et al*. (19) 7.63 ± 7.49, Kurt *et al*. (23) 9.95 ± 10.42 in the control groups of their study. Based on these results; It has been commented that values higher than 3% may occur due to the shape, angular and spatial differences between the right and left condyles and can be accepted as normal (19). In our study, the CAI was measured as 11.6 ± 14.21 in the study group and a statistically significant difference was found between the control group (*p* = 0.047). In addition, the RAI was measured as 1.30 ± 1.03 in the control group and 2.07 ± 1.64 in the study group, and again a statistically significant difference was found between them (*p* = 0.016).

The most common odontogenic cyst is the radicular cyst, followed by the dentigerous cyst, and in various demographic studies it was found that it was seen in 65.25% and 24.08% (24), 66.4% and 19.2% (25) of the cysts, respectively. Radicular cysts are caused by the cystic degeneration of Malassez epithelial remnants in the periapical region by the inflammatory stimulus of pulpal infection (5). In other words, there is a tooth associated with an endodontic lesion (apical periodontitis) in the etiology of radicular cysts (26). Endodontic lesions are painful and the involved tooth is sensitive. This situation may cause the patient to avoid chewing with the painful side and to develop unilateral chewing habit in the patient (27). On the other hand, teeth associated with radicular cysts often have large caries and fractures that disrupt the occlusal balance. Dental caries, early tooth loss and trauma are among the possible environmental causes of asymmetries in the face and dental arch (19).

In addition, radicular cysts and dentigerous cysts may cause expansion of one or both cortical bones and when they reach large sizes, they can cause tooth displacement and root resorption (5). These may have caused the development of condylar and ramal asymmetry, which we detected in the study, by changing the chewing pattern (6,7) and force balance of the patient over time. As a matter of fact, according to the functional and mechanical hypothesis (28) used to explain the asymmetry, there is a relationship between the load on the joint and the condyle size (11).

## Conclusions

No statistically significant difference was observed between any of the variables of asymmetry according to gender. CAI and RAI values were higher in the study group compared to the control group, and this difference was statistically significant.

In our study, we found that the presence of intraosseous cysts larger than 10 mm in the jaws is associated with condylar and ramal asymmetry. Condylar asymmetry is also a diagnostic criterion for TMD (11,20). Therefore, in our next study, we planned to evaluate the relationship between TMD and intra-bone cysts in the jaws.
